# Microarray analysis demonstrates up-regulation of the endothelin-1 gene with compensatory down-regulation of the ET_A_ receptor gene in human portal vein

**DOI:** 10.1042/BSR20240528

**Published:** 2024-07-05

**Authors:** Nicola E. Owen, Thomas L. Williams, Janet J. Maguire, Rhoda E. Kuc, Emma E. Davenport, Anthony P. Davenport

**Affiliations:** 1 Experimental Medicine and Immunotherapeutics, Department of Medicine, University of Cambridge, CB2 0QQ, U.K.; 2 Wellcome Sanger Institute, Wellcome Genome Campus, Cambridge, CB10 1SA, U.K.

**Keywords:** cirrhosis, Endothelin, ETA receptor, ETB receptor, portal hypertension

## Abstract

High blood pressure in the portal vein, portal hypertension (PH), is the final common pathway in liver cirrhosis regardless of aetiology. Complications from PH are the major cause of morbidity and mortality in these patients. Current drug therapy to reduce portal pressure is mainly limited to β-adrenergic receptor blockade but approximately 40% of patients do not respond. Our aim was to use microarray to measure the expression of ∼20,800 genes in portal vein from patients with PH undergoing transplantation for liver cirrhosis (PH, *n*=12) versus healthy vessels (control, *n*=9) to identify potential drug targets to improve therapy. Expression of 9,964 genes above background was detected in portal vein samples. Comparing PH veins versus control (adjusted *P-*value < 0.05, fold change > 1.5) identified 548 up-regulated genes and 1,996 down-regulated genes. The 2,544 differentially expressed genes were subjected to pathway analysis. We identified 49 significantly enriched pathways. The endothelin pathway was ranked the tenth most significant, the only vasoconstrictive pathway to be identified. ET-1 gene (*EDN1*) was significantly up-regulated, consistent with elevated levels of ET-1 peptide previously measured in PH and cirrhosis. ET_A_ receptor gene (*EDNRA*) was significantly down-regulated, consistent with an adaptive response to increased peptide levels in the portal vein but there was no change in the ET_B_ gene (*EDNRB*). The results provide further support for evaluating the efficacy of ET_A_ receptor antagonists as a potential therapy in addition to β-blockers in patients with PH and cirrhosis.

## Introduction

In humans the portal vein delivers partially deoxygenated blood from the mesenteric and splenic circulation to the liver [1], with the remaining 25% supplied from the hepatic artery. The portal vein branches into a network of smaller vessels (sinusoids) and travels through the liver to supply nutrients. The portal system is a low pressure, low resistance system, and the normal portal vein pressure varies between 5 and 10 mmHg [2].

Cirrhosis is the most common cause of increased portal blood pressure, resulting in portal hypertension (PH), and is a leading cause of death worldwide. Compensated cirrhosis, where PH complications have not yet developed, is estimated to affect 112 million people worldwide, corresponding to an age-standardised global prevalence of 1,395 cases per 100,000 population. The number of cases of decompensated cirrhosis, where severe complications of PH have developed, is increasing worldwide and was estimated at 10.6 million globally, with an age-standardised global prevalence of 132.5 per 100,000 population [3,4].

The hepatic venous pressure gradient (HVPG) is measured clinically to estimate the pressure in the portal vein. It encompasses the pressure gradient between the portal and the hepatic veins, and an HVPG of > 5 mmHg defines PH. The two well-characterised pathological components of PH are increased intrahepatic vascular resistance and splanchnic vasodilation [5]. The increase in intrahepatic vascular resistance in liver cirrhosis (severe scarring of the liver) is a result of progressive, fibrotic architectural disruption of the hepatic anatomy, due to fibrosis and the increased vascular tone within the portal vein and liver, secondary to an imbalance in vasoconstrictors and vasodilators. The increase in intrahepatic resistance leads to splanchnic vasodilation and diversion of blood flow through portosystemic collaterals, which further worsens PH.

High blood pressure in the portal vein, is the final common pathway in liver cirrhosis, as the liver attempts to repair itself, regardless of aetiology [5]. These include metabolic dysfunction-associated steatotic liver disease (MASLD) caused by build-up of fat in the liver as a result of obesity, metabolic syndrome or Type 2 diabetes; chronic infection by viruses (hepatitis C or hepatitis B); alcohol-related liver disease (ARLD); and conditions that cause inflammation in the bile ducts (primary biliary cholangitis and primary sclerosing cholangitis). The increased pressure may lead to the development of large, swollen veins (varices) in the gut that can rupture, causing gastrointestinal bleeding, and ascites, the accumulation of fluid in the abdomen, causing swelling. Complications from PH are the major cause of morbidity and mortality in these patients. HVPG needs to increase above a critical threshold value of 10 mmHg to be associated with clinical manifestation of the complications of PH [6]. Maintaining portal pressure below this threshold by therapeutic interventions prevents the complications of PH.

Current treatment options for PH are very limited. Lifestyle changes such as abstaining from alcohol and performing more aerobic exercise can moderately improve the architectural hepatic damage that underlies PH [7,8]. Liver transplantation is also an effective treatment for PH, but recipient demand exceeds donor organ availability [9]. Pharmacological treatment typically [8,10] includes splanchnic vasoconstrictors to reduce portal inflow [11]. Intravenous terlipressin (a vasopressin analogue) [12], somatostatin, or somatostatin analogues (such as octreotide) [13,14] are given as short-term therapeutic options, primarily in the acute setting to treat variceal haemorrhage. Targeting the portal vein, current drug therapy for long-term treatment of PH is limited to non-selective β-adrenoceptor blockers such as propranolol [15], nadolol [16], and carvedilol (which is additionally an α_1_-blocker) [17,18] to decrease portal pressure. However, ∼40% of patients fail to respond [19]. There is a current unmet need to identify new drugs to improve therapeutic options, particularly in patients where PH is not controlled by β-blockers.

A number of endogenous vasoactive molecules, including angiotensin II (Ang II), have been shown to regulate the flow of blood through the liver. In addition, Ang II is thought to play a major role in hepatic fibrosis [20], as well as mediating other actions (inflammatory cytokines, mitogenesis, proliferation, and collagen synthesis). Endothelin-1 (ET-1) is one of the most potent constrictors of human vessels, characterised by long-lasting constrictor actions and is an attractive potential new agent contributing to PH. ET-1 is a cleavage product of the precursor prepro-ET-1 [21–24], released from endothelial cells to exert its constrictor actions mainly through ET_A_ receptors expressed on vascular smooth muscle cells [25,26]. Plasma ET-1 levels, and those of a second endogenous isoform, ET-3, are significantly elevated in patients with chronic liver disease and PH [27–32], and levels are increased in cirrhotic liver tissue where endothelin peptides are hypothesised to contribute to disease pathogenesis. Further, ET-1 binding sites have been identified in hepatic sinusoids and the portal vein in rats [33], and ET-1 levels are elevated in rat models of liver cirrhosis [24,34], contributing to PH. ET-1-induced vasoconstriction was shown to cause a significant increase in portal pressure of the rat liver via a localized constrictive effect on the distal segment of preterminal portal venules, and contributed to PH [35,36].

ET_A_ antagonism has been shown to reduce portal pressure, increase sinusoidal vasodilatation, and reduce hepatic fibrosis in animal models of cirrhosis and PH, providing proof-of-principle for this class of drug as a potential therapeutic option in this disease area [37–41]. Despite a wealth of pre-clinical evidence, to date, no ET antagonist has been approved in PH.

Identifying genes that are differentially expressed between healthy and diseased human tissue is emerging as a robust strategy to identify new drug targets and confirm results from pre-clinical animal models to better understand the pathology of diseases. Genetic data are a key criterion required by pharmaceutical companies for target validation and has been shown to reduce failure rates in clinical trials. One of the most clinically relevant strategies is the identification of differentially expressed genes in disease encoding proteins, particularly where there are already approved therapeutic or investigational agents that can be tested for repurposing in clinical trials. This strategy can be further enhanced using *in vitro* pharmacology assays to measure functional responses of proteins encoded by the top differentially expressed genes to confirm expression of the drug target in diseased human tissue.

Our aim was to use microarray to measure the expression of ∼20,800 genes in portal vein from patients with PH undergoing transplantation for liver cirrhosis versus healthy vessels. This strategy has not previously been applied to blood vessels. Genes were identified as significantly up- or down-regulated in PH tissue to define a landscape for the disease. These differentially expressed genes were subjected to pathway analysis to identify potential drug targets. The ET system was the only vasoconstrictor pathway identified as being significantly enriched in cirrhotic portal vein compared with controls.

## Methods

### Tissue acquisition and ethics

Surgical samples of portal vein were obtained at the time of liver transplantation from 12 patients (9 males, 3 females, age = 54.3 ± 2.0), with cirrhosis (Table 1) caused by a range of pathologies but with clinically significant PH, recognised as the final pathway of chronic liver disease regardless of aetiology [5]. Control samples (*n*=9) were from healthy donor livers (7 males, 2 females).

**Table 1:** Demographics of patients with cirrhosis donating portal veins (*n*=12)

Age	Sex	Aetiology	Blood results						Varices grade	Ascites	HE	UKELD	β-Blocker
			Na	Alb	Cr	Bil	PT	INR					
55	M	ARLD/MASLD	133	30	169	42	16.5	1.4	2	3	0	57	Y
45	F	HCV	124	30	50	75	18.7	1.6	3	3	0	64	N
63	M	HCV/ARLD	137	24	71	39	18.0	1.5	3	2	1	54	N
53	M	ARLD	134	27	49	115	29.1	2.5	2	3	1	61	N
63	M	ARLD	132	26	74	40	16.9	1.4	3	3	1	56	Y
61	M	MASLD	145	25	83	56	20.7	1.8	3	3	0	51	Y
48	F	HCV	130	26	73	8	12.8	1.1	0	3	0	51	Y
48	M	PSC	131	22	65	193	19.2	1.6	1	2	0	63	N
48	M	PSC	132	20	55	23	13.7	1.2	3	3	0	53	N
48	M	AIH	142	26	51	32	17.1	1.4	3	3	0	49	Y
64	M	ARLD	138	26	85	106	20.7	1.7	2	1	0	57	N
56	F	ARLD	135	25	84	22	20.0	1.7	2	2	0	54	Y

Abbreviations: AIH, autoimmune hepatitis; Alb, albumin (g/l); ARLD, alcohol-related liver disease; Asc, ascites; Bil, bilirubin (μmol/l); Cr, creatinine (μmol/l); HCV, hepatitis C virus; INR, international normalised ratio; MASLD, metabolic dysfunction-associated steatotic liver disease; Na, sodium (mmol/l); Plt, platelets (10 × 9/l); PSC, primary sclerosing cholangitis; PT, prothrombin time (s). Blood results were derived from the last samples taken prior to transplant. Scoring systems: Varices: Italian Liver Cirrhosis Project Classification (ILCP) [72] - 1: small <25% of the lumen, 2: 25–50% of the lumen, 3: large, >50% of the lumen, Ascites: Child-Pugh Score for ascites - 1: none, 2: mild (or suppressed with medication), 3: moderate-to-severe (or refractory), Hepatic encephalopathy (HE) using West Haven criteria - 0: none, 1: changes in behaviour with minimal change in the level of consciousness, 2: gross disorientation, drowsiness, inappropriate behaviour, 3: marked confusion, incoherent speech, sleeping but rousable to vocal stimuli, 4: comatose.

All samples were obtained with ethical approval (REC reference 10/H0305/33) and written informed consent. The specific location of the portal vein tissue sampling was determined by ease of sampling and was used to represent the portal venous vasculature. Portal vein samples were approximately 1.5 cm in length, taken as excess from donor livers, and which would otherwise have been discarded. Surgeons aimed to consistently take samples below the bifurcation into the left and right portal vein branches, at the level of the hilum where the portal vein enters the liver, to ensure that as similar an area as possible was sampled across all specimens. Once collected, samples were frozen in liquid nitrogen immediately and stored at −80 °C.

### RNA extraction

On the day of experimentation, vessel tissue ∼0.5 cm^2^ was taken directly from the -80°C freezer and chopped finely, whilst kept on ice, and placed into ceramic bead lysing matrix tubes (MP Biomedicals). RNA was extracted using the RNeasy Plus Universal Mini Kit (QIAGEN, Hilden, Germany) according to the manufacturer’s instructions, with an additional DNAse treatment step.

RNA extraction was assessed for concentration and quality using a SpectroStar (BMG Labtech, Aylesbury, U.K.) and a Bioanalyser (Agilent Technologies, Cheadle, U.K.). Microarray experiments were performed at Cambridge Genomic Services, University of Cambridge, using a species-specific Clariom S Human HT Array Plate (Affymetrix, Wooburn Green, U.K.), which measures the expression of ∼20,800 genes using ∼205,800 oligonucleotide probes, length 25 bp with usually 10 probes per gene.

In addition, a small number of genes in this microarray are measured and recorded more than once. We therefore report the total number of probes which is slightly higher than the number of genes. However, we followed convention and all probes were used in the analysis.

Total RNA (50 ng) was amplified along with inline PolyA spike in control RNA, using the GeneChip Pico Reagent Kit that enables the highly sensitive detection in the small volume tissue samples of portal vein. Successfully amplified samples were labelled using the Pico WT terminal labelling kit using the inline hybridization controls. Samples were hybridized to the array, washed, stained and scanned using GeneTitan instrumentation. All reagents were from Affymetrix and used according to the manufacturer’s instructions.

### Quality control

A series of quality control tests were carried out. These included:

1. Normalised Unscaled Standard Error values calculated by fitting a model to the probe-intensity data and the standard error of the model was used to check the sample quality. Distributions were expected to be centred near 1, whereas an array with a boxplot centred around 1.1 or higher was identified as poor quality.
2. Density plots of the distribution of intensities versus log-frequency.
3. A pseudo sample constructed using the intensity of all the samples. The log ratio of intensity between the sample and the pseudo sample was plotted versus the mean average of intensity of each sample. Deviations from the central line of the pseudo sample were used to identify poor quality samples.

In further quality control, the relationships between the samples were assessed. Principal component clustering showed that the 12 patients with cirrhosis and portal hypertension all clustered into one group, distinct from the healthy vessels (control, *n*=9). If the contribution of different aetiologies/causes of liver cirrhosis was significant, it might be expected that patients with portal hypertension would cluster into more than one group, but this was not the case in our study. This step in the quality control provided the rationale for including patients with a range of aetiologies, as the focus was on measuring mRNA encoding genes in the portal vein, specifically to identify vasoconstrictor pathways from the portal blood vessels for possible new drug interventions.

### Data analysis

The data were background corrected, normalised using the Robust Multichip Analysis (RMA) method [42], and summarised. Once the data were processed, the comparisons between control and PH (cirrhotic) portal vein were performed using the R limma package [43], and the results were corrected for multiple testing using the False Discovery Rate (FDR) [42] with a threshold of an adjusted *P-*value < 0.05.

## Identification of drug targets

### Pathway analysis

Individual genes that were significantly differentially expressed with an adjusted *P-*value < 0.05 and a biologically meaningful fold change (FC) > 1.5 (corresponding to log_2_FC > 0.58 for up-regulated or log_2_FC < -0.58 for down-regulated genes). Comparisons of individual genes in the ET pathway and genes encoding targets of current approved drugs used in treating PH were compared by *t*-test (*P-*value < 0.05).

Pathway analysis of the differentially expressed genes was carried out using eXploring Genomic Relations (XGR) (http://galahad.well.ox.ac.uk/XGR, Wellcome Trust Centre for Human Genetics, University of Oxford) for enhanced interpretation of genomic summary data [45]. Principal gene(s) in these pathways were compared against a curated list of genes encoding proteins to identify further targets in PH from the GuidetoPharmacology database (https://www.guidetopharmacology.org/) [46].

### *In vitro* pharmacology

Experiments were carried out as previously described [47]. Briefly, portal vein tissues from one transplant surgery (*n*=2) were cut into 3 × 4 mm rings and set up in organ baths, in modified Krebs solution at 37°C. Responses to potassium chloride (KCl) were used to check viability of the tissue and to measure the maximal vasoconstriction response at the end of the experiment. Concentration-response curves were constructed to ET-1 (10^−10^ to 10^−6^ M) in the presence or absence of the endothelin receptor antagonist ambrisentan (3 μM) that was added to the bath 30 min before addition of ET-1. Responses to ET-1 were expressed as a percentage of the terminal KCl response, and data were analysed to determine values of EC_50_ (the concentration producing half-maximal response) for ET-1 in the donor and PH veins and to estimate the potency (pA_2_, the -log_10_ concentration of the antagonist required to produce a 2-fold shift of the agonist concentration–response curve) of ambrisentan to block ET-1 vasoconstriction. Data were analysed using Prism 6 (Graph-Pad Software Inc, San Diego, CA).

## Results

Characteristics of patients with cirrhosis and PH donating portal vein samples are given in Table 1. The median age was 54 years, interquartile range 48–62 years, 9 males, 3 females, age = 54.3 ± 2.0. The mean United Kingdom Model for End-Stage Liver Disease (UKELD) score was 56.3 ± 1.2, a measure of liver disease severity and radiological evidence of portal hypertension. In the United Kingdom, patients with scores ≥ 49 are considered for transplantation. It was originally derived in 2008 from the Model for End-Stage Liver Disease (MELD) score, incorporating the serum sodium level. Demographics of healthy control liver sample donors are provided in Table 2 (7 males, 2 females, median age: 48, interquartile range 25–63).

**Table 2:** Demographics of individuals donating control portal veins (*n*=9)

Age	Sex
52	M
66	M
19	F
66	M
60	M
17	M
30	F
43	M
48	M

Clinically significant portal hypertension (CSPH) in cirrhosis is defined as an HVPG of 10 mmHg or greater. The complications of portal hypertension can arise at this point, including the formation of varices and development of ascites. HVPG is not always routinely measured in the U.K., owing to its invasiveness and technical difficulty. There are emerging non-invasive ways of assessing the degree of CSPH recognized by its clinical signs and symptoms [48]. All patients in the present study had evidence of CSPH such as combinations of varices, variceal bleeding, hepatic encephalopathy or ascites.

**Figure 1:**
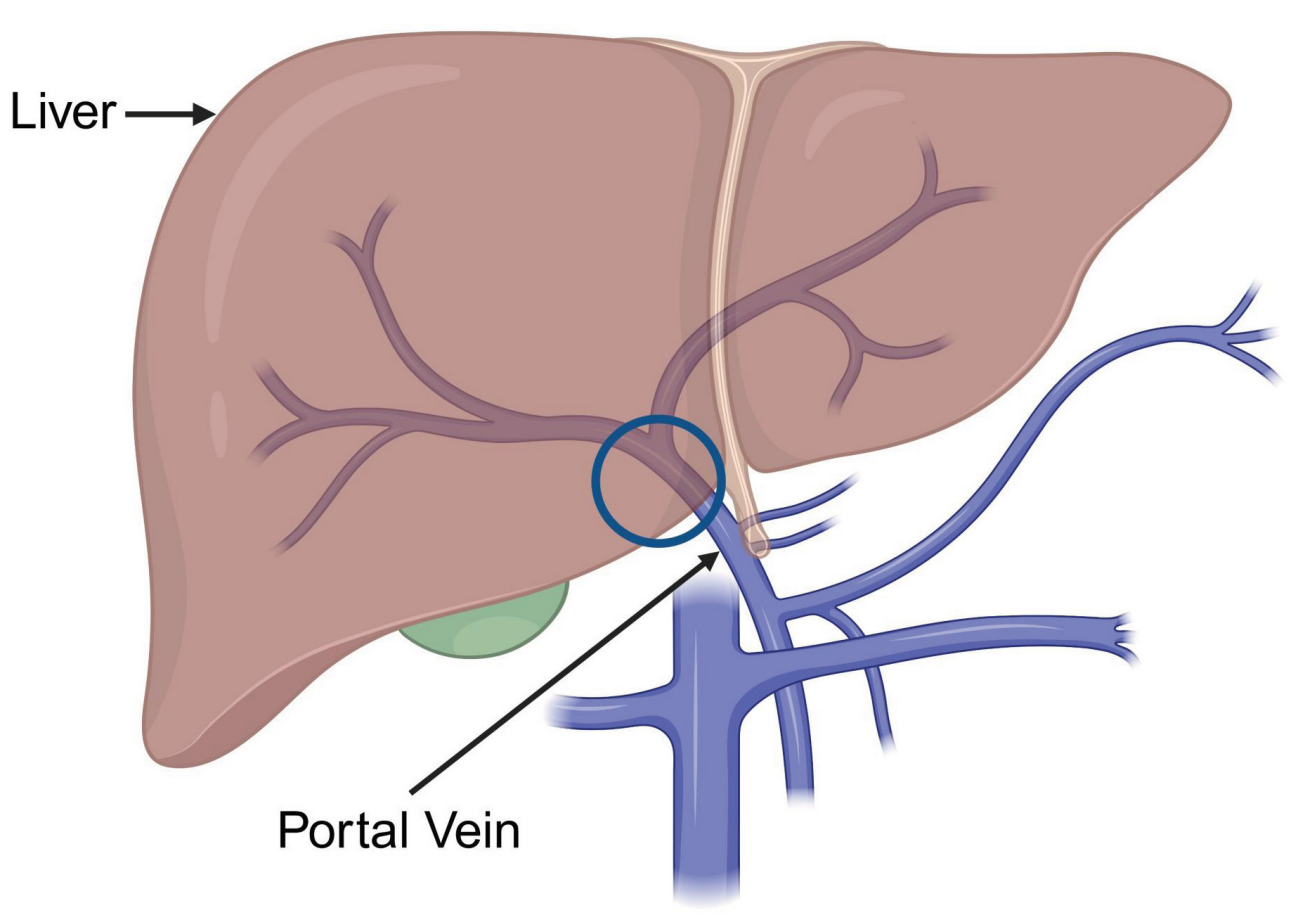
Diagram showing location of main portal vein Tissue samples were taken from the main portal vein where surgeons aimed to consistently remove tissue from below the bifurcation into the left and right portal vein branches, at the level of the hilum where the portal vein enters the liver (indicated by the circle), to ensure that as similar an area as possible was sampled across all specimens. Samples contained predominantly smooth muscle and endothelial cells. Created with BioRender.com.

**Figure 2:**
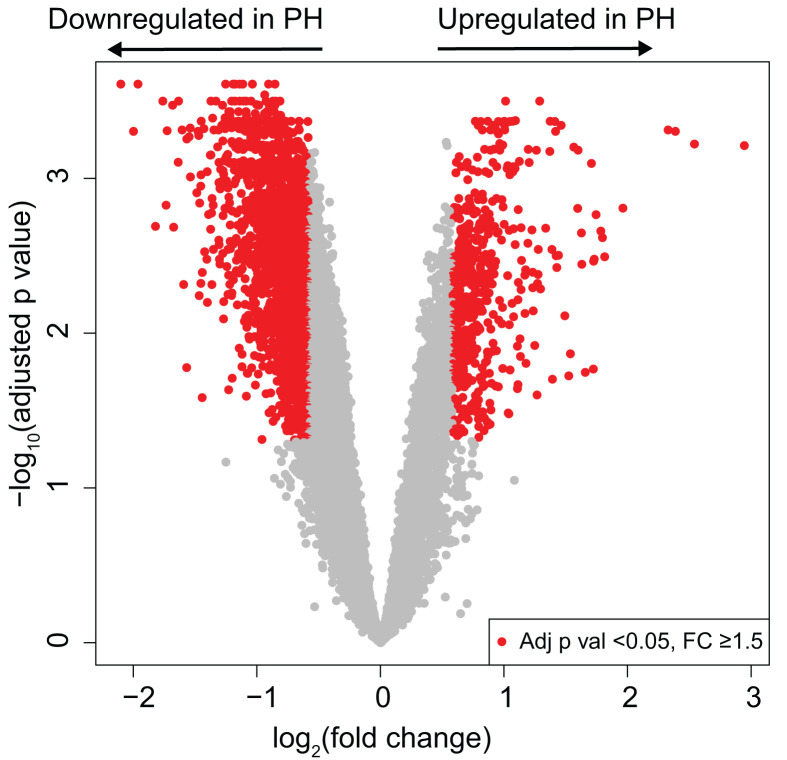
Differential gene expression in portal vein from patients with portal hypertension (PH) Volcano plot demonstrating differentially expressed genes in portal vein from patients transplanted for cirrhosis (PH) versus control vein. Red colouring shows adjusted *P-*value < 0.05 and fold change > 1.5 for genes.

The portal vein (Figure 1) comprises predominantly smooth muscle and endothelial cells and the genes expressed are likely to be expressed mainly by these cell types. Following filtration of probes to remove intensities in the background range, expression of 9,964 genes (measured with 10,446 probes) in the portal vein samples was detected above background. Comparing control versus PH portal vein, 2,544 genes (2,598 probes) were differentially expressed (adjusted *P-*value < 0.05 and FC > 1.5). 1,996 genes (2,038 probes) were down-regulated; 548 genes (560 probes) were up-regulated (Figure 2).

### Pathway analysis identified the ET pathway as being significantly enriched in cirrhotic portal vein compared with controls

To investigate the functional role, pathway analysis of the 2,544 differentially expressed genes, using XGR (http://galahad.well.ox.ac.uk/XGR), identified 49 pathways that were significantly enriched (adjusted *P-*value < 0.05). The results are summarised in Table 3.

**Table 3:** Pathway analysis using XGR (http://galahad.well.ox.ac.uk/XGR) identified 49 significant pathways (adjusted *P*-value < 0.05 and FC > 1.5 (log_2_FC of > 0.58 or < −0.58)) together with  *Z* scores

	Pathway	Proposed function	*Z* score	Adjusted *P* value	Number of genes in pathway
1	IL6-mediated signalling events	Hepatocyte mitogen	5.11	0.0003	18
2	AP-1 transcription factor network	AP-1 promotes liver fibrosis	4.98	0.0003	23
3	Validated transcriptional targets of AP1 family members Fra1 and Fra2	AP1 is a proposed modulator of metabolic liver diseases	5.07	0.0003	15
4	Genes related to regulation of the actin cytoskeleton	Activation of hepatic stellate cells in fibrosis	4.34	0.0024	13
5	Calcineurin-regulated NFAT-dependent transcription in lymphocytes	Control of immune function	4.25	0.0024	16
6	HIF-1α transcription factor network	Adaptive response to hypoxia	4.14	0.0025	20
7	Plasma membrane estrogen receptor signalling	Estrogens protective in liver disease	3.99	0.004	14
8	Trk receptor signalling mediated by the MAPK pathway	Cell proliferation	3.83	0.0059	12
9	IL4-mediated signalling events	Switch for macrophage polarization in progression of liver fibrosis	3.71	0.0063	18
10	Endothelins	Vasoconstriction	3.63	0.0072	18
11	Differentiation pathway in PC12 Cells; this is a specific case of PAC1 receptor pathway	PC12 proliferation and differentiation	3.58	0.0083	14
12	Sphingosine-1-phosphate receptor 3 (S1P3) pathway	Proliferation, differentiation, vascular angiogenesis, vasodilatation	3.54	0.0092	10
13	PAR1-mediated thrombin signalling events	Thrombotic coagulation response, endothelial barrier integrity, inflammation	3.42	0.011	13
14	Reelin signalling pathway	Extracellular matrix and migration	3.41	0.011	10
15	alidated targets of C-MYC transcriptional repression	Transcription factor	3.34	0.011	17
16	C-MYB transcription factor network	Transcription factor	3.23	0.014	21
17	Notch signalling pathway	Cell–cell communication, angiogenesis, and is implicated in liver disease in Alagille syndrome	3.19	0.015	16
18	IFN-γ pathway	Inflammation (including hepatic), migration and proliferation of vascular smooth muscle cells	3.16	0.015	12
19	Integrin-linked kinase signalling	Cell migration, proliferation, and adhesion, blood vessel integrity and neovascularisation	3.13	0.015	13
20	IL2-mediated signalling events	Immune response, vascular permeability	3.11	0.015	15
21	IL3-mediated signalling events	Immune response, vascular smooth muscle migration and proliferation	3.11	0.015	9
22	Granule cell survival pathway is a specific case of more general PAC1 receptor pathway	Granule cell survival	3.11	0.015	9
23	CXCR4-mediated signalling events	Chemotactic activity for lymphocytes, angiogenesis and vascularisation of tumour vessels, implicated in liver disease	2.98	0.018	23
24	GMCSF-mediated signalling events	Cytokine, stem cell differentiation to granulocytes	2.99	0.019	11
25	N-cadherin signalling events	Cell–cell adhesion, vascular wall integrity	2.92	0.021	10
26	Calcium signalling in the CD4+ TCR pathway	Inflammatory/immune response	2.86	0.023	9
27	Signalling events mediated by VEGFR1 and VEGFR2	Vasculogenesis/angiogenesis, vascular permeability, potential vasodilatation	2.84	0.023	17
28	Signalling events mediated by hepatocyte growth factor receptor (c-Met)	Cancer angiogenesis and cell invasiveness, hepatocyte regeneration	2.82	0.023	19
29	EPO signalling pathway	Red blood cell production, implicated in hypertension	2.81	0.023	10
30	ATF-2 transcription factor network	Activating transcription factor	2.8	0.023	15
31	Signalling events mediated by focal adhesion kinase	Regulates hepatic stellate cell activation and liver fibrosis	2.8	0.023	15
32	Genes related to chemotaxis	Chemotaxis	2.78	0.023	12
33	CDC42 signalling events	Cell morphology, cell migration, and cell cycle progression, implicated in liver regeneration	2.77	0.023	17
34	Glucocorticoid receptor regulatory network	Regulator of transcription	2.76	0.023	19
35	Sphingosine-1-Phosphate (S1P1) pathway	Immune response, vasculogenesis, vasodilatation, protective role in the liver	2.74	0.023	7
36	Coregulation of androgen receptor activity	Regulator of transcription	2.73	0.024	15
37	LPA receptor mediated events	Implications in inflammation, thrombosis and atherosclerosis	2.68	0.026	16
38	Nongenotropic androgen signalling	Implications in hepatocellular carcinoma, cirrhosis, and hepatitis	2.62	0.029	9
39	SHP2 signalling	Protects endothelial barrier, indirect role in vasodilatation through activation of eNOS	2.56	0.033	14
40	IL5-mediated signalling events	Immune response, B-cell growth and differentiation, vascular cell growth and migration	2.5	0.033	5
41	HIF-2α transcription factor network	Angiogenesis, regulates hepatocyte cell proliferation during hepatic outgrowth	2.51	0.033	9
42	Syndecan-2-mediated signalling events	Cell proliferation, cell migration and cell–matrix interactions, vascular permeability	2.51	0.033	9
43	Validated targets of C-MYC transcriptional activation	Transcription factor	2.45	0.038	17
44	Fas signalling pathway	Programmed cell death, role in liver cirrhosis transition to hepatocellular carcinoma	2.45	0.038	15
45	Role of Calcineurin-dependent NFAT signalling in lymphocytes	Immune response	2.38	0.042	13
46	DNA-PK pathway in nonhomologous end joining	Potential role in alcohol-related liver disease	2.32	0.042	5
47	Sumoylation by RanBP2 regulates transcriptional repression	Nuclear pore protein	2.28	0.042	4
48	STAT3 pathway	Transcription factor, regulator of liver fibrosis	2.28	0.042	4
49	Direct p53 effectors	Tumour suppressor gene, with potential roles in vascular remodelling and atherosclerosis	2.33	0.045	27

All values are above 2 indicating at least two standard deviations from the mean.

Many of the identified pathways play a role in inflammatory/immune responses, encompassing signalling events mediated by interleukins (IL-2,3,4,5, and 6) and interferon-γ, amongst others. Other pathways comprise transcriptional regulators and networks, such as HIF-1α and HIF-2α, AP-1, ATF-2 and STAT3, and regulators of cell morphology, proliferation, differentiation, adhesion, migration, and cell–cell interaction.

Of the 49 pathways, several are potentially directly involved in liver fibrosis, cirrhosis, alcohol-related liver disease, and liver regeneration, including pathways associated with the AP-1 network, estrogen/androgen signalling pathways, and the CXCR4 receptor.

A number of the pathways are implicated in vasculogenesis, angiogenesis, vascular wall integrity, and vessel permeability, including PAR-1, syndecan-2, and VEGFR1/VEGFR2 signalling events. The SHP2 signalling pathway may cause phosphorylation of eNOS in endothelial cells to induce vasodilatation [49]. The sphingosine-1-phosphate pathway, through activation of the sphingosine-1-phosphate receptor 3 (S1P3), can also mediate vascular tone, inducing vasodilatation through endothelial cell eNOS [50–52].

**Figure 3:**
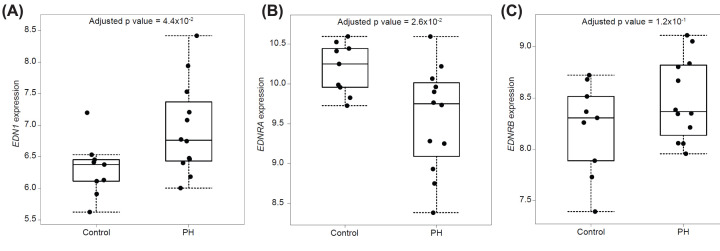
Comparison of differentially expressed genes in the ET pathway in cirrhotic portal vein (PH) versus control Expression of (**A**) the ET-1 gene (*EDN1*) was significantly up-regulated, consistent with elevated levels of ET-1 peptide previously measured in cirrhosis. (**B**) ET_A_ (*EDNRA*) receptor was down-regulated, likely to be present on smooth muscle and consistent with an adaptive response to increased peptide levels in the portal vein. There was no significant change in (**C**) ET_B_ (*EDNRB*), consistent with retention of ET_B_ mediated vasodilatation in these vessels.

The endothelin pathway was ranked the tenth most significantly differentially expressed (adjusted *P-*value = 0.0072), and was the only strictly vasoconstrictive pathway identified in the top 49 enriched pathways (Figure 3). Expression of the ET-1 gene (*EDN1*) was significantly up-regulated (adjusted *P-*value = 4.4 × 10^−2^, ln FC 0.63) (Figure 3A), consistent with elevated levels of ET-1 peptide previously measured in cirrhosis. Expression of ET_A_ receptor gene (*EDNRA*) was significantly down-regulated (adjusted *P-*value = 2.6 × 10^−2^ ln FC −0.62) (Figure 3B), likely to be present on smooth muscle and consistent with an adaptive response to increased peptide levels in the portal vein. There was no significant change in ET_B_ (*EDNRB*) (Figure 3C), consistent with retention of ET_B_-mediated vasodilatation in these vessels. mRNA encoding endothelin-converting enzyme-1 (*ECE-1*), the principal enzyme catalysing conversion of the biologically active peptide from the inactive precursor, was unaltered. Genes encoding the two other isoforms ET-2 (*EDN2*) and ET-3 (*EDN3*) were not detected.

### Vasoconstrictor receptors in control and PH portal vein

**Figure 4:**
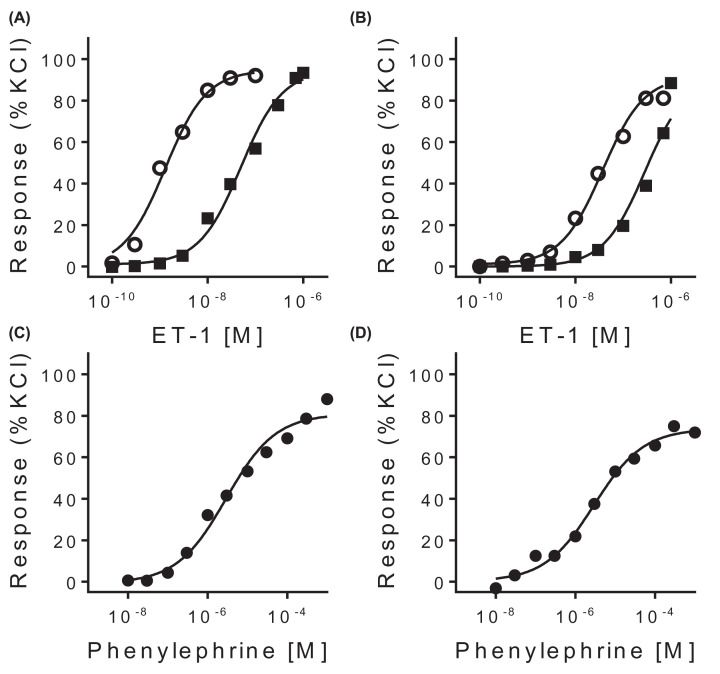
*In vitro* pharmacology of isolated portal veins Concentration–response curves for ET-1 in the absence (○) and presence (■) of 3 µM ambrisentan in (**A**) control and (**B**) PH portal vein. Concentration-response curves for phenylephrine (●) in (**C**) control and (**D**) PH portal vein.

Despite down-regulation of the gene encoding ET_A_, in preliminary experiments ET-1 contracted both control (Figure 4A) and PH (Figure 4B) portal veins, although higher concentrations of endothelin were required to elicit a response in the PH portal vein (EC_50_ = 30.4 nM) compared with the control portal vein (EC_50_ = 1.17 nM), consistent with the down-regulation of the ET_A_ receptor and increased levels of ET-1 observed in the disease. Importantly, the ET_A_ receptor antagonist ambrisentan competitively blocked the response to ET-1 to a similar extent, with a pA_2_ = 7.07 in the control portal vein compared with pA_2_ = 6.48 in the PH vessel, validating the ET_A_ receptor as a target in PH. Phenylephrine, an α-1 adrenoceptor agonist, also contracted the control (Figure 4C) and PH (Figure 4D) veins suggesting the presence and viability of these receptors to cause vasoconstriction in these vessels. Potency of phenylephrine was comparable in control portal vein (EC_50_ = 3.1 µM) and PH vein (EC_50_ = 3.2 μM).

### Expression of genes for target proteins of current approved agents in portal hypertension

Of the 49 significantly enriched pathways, we identified 14 that are targeted by currently approved agents indicated in other disease areas, which could provide further targets in PH and opportunities for repurposing. These findings are summarised in Table 4.

**Table 4:** Identification of new therapeutic targets in PH

	Pathway	Proposed function	Gene(s) encoding the principal target protein	Examples of approved drugs targeting the protein(s) Therapeutic class
1	IL6-mediated signalling events	Hepatocyte mitogen	*IL6*	Siltuximab (Monoclonal antibody)
2	AP-1 transcription factor network	AP-1 promotes liver fibrosis	*JUNB*	
3	Validated transcriptional targets of AP1 family members Fra1 and Fra2	AP1 is a proposed modulator of metabolic liver diseases	*JUNB*	-
4	Genes related to regulation of the actin cytoskeleton	Activation of hepatic stellate cells in fibrosis		-
5	Calcineurin-regulated NFAT-dependent transcription in lymphocytes	Control of immune function	*NFATC1 NFATC2 NFATC3*	-
6	HIF-1α transcription factor network	Adaptive response to hypoxia	*HIF1A*	-
7	Plasma membrane estrogen receptor signalling	Estrogens protective in liver disease	*ESR1* *ESR2*	Diethylstilbestro (Synthetic agonist)
8	Trk receptor signalling mediated by the MAPK pathway	Cell proliferation	*TRK*	-
9	IL4-mediated signalling events	Switch for macrophage polarization in progression of liver fibrosis	*IL4* *IL4R*	-
10	Endothelins	Vasoconstriction	*EDNRA*	Clazosentan (Synthetic antagonist)
11	Differentiation Pathway in PC12 Cells; this is a specific case of PAC1 (pituitary adenylate cyclase activating polypeptide 1) Receptor Pathway.	PC12 proliferation and differentiation	*ADCYAP1R1*	
12	Sphingosine-1-phosphate receptor 3 (S1P3) pathway	Proliferation, differentiation, vascular angiogenesis, vasodilatation	*S1PR3*	
13	PAR1 (protease-activated receptor 1)-mediated thrombin signalling events	Thrombotic coagulation response, endothelial barrier integrity, inflammation	*F2R*	Vorapaxar (Synthetic antagonist)
14	Reelin signalling pathway	Extracellular matrix and migration	*RELN*	
15	Validated targets of C-MYC transcriptional repression	Transcription factor	*MYC*	
16	C-MYB transcription factor network	Transcription factor	*MYB*	
17	Notch signalling pathway	Cell–cell communication, angiogenesis, and is implicated in liver disease in Alagille syndrome	*Notch1*	
18	IFN-γ pathway	Inflammation (including hepatic), migration and proliferation of vascular smooth muscle cells	*IFNG* *IFNGR1* *IFNGR2*	Emapalumab (Monoclonal antibody)
19	Integrin-linked kinase signalling	Cell migration, proliferation, and adhesion, blood vessel integrity and neovascularisation	*ILK*	
20	IL2-mediated signalling events	Immune response, vascular permeability	*IL2* *IL2A* *IL2B* *IL2G*	IL2 peptide (agonist) Daclizumab, (Monoclonal antibody)
21	IL3-mediated signalling events	Immune response, vascular smooth muscle migration and proliferation	*IL3* *IL3RA* *IL3RB*	
22	Granule Cell Survival Pathway is a specific case of more general PAC1 Receptor Pathway.	Granule cell survival	*ADCYAP1R1*	
23	CXCR4-mediated signalling events	Chemotactic activity for lymphocytes, angiogenesis and vascularisation of tumour vessels, implicated in liver disease	*CXCR4* *CXCL12*	Ulocuplumab (Monoclonal antibody)
24	GMCSF-mediated signalling events	Cytokine, stem cell differentiation to granulocytes	*GMCS2* *CSF2RA*	G-CSF (Peptide agonist)
25	N-cadherin signalling events	Cell–cell adhesion, vascular wall integrity		
26	Calcium signalling in the CD4+ TCR pathway	Inflammatory/immune response		
27	Signalling events mediated by VEGFR1 and VEGFR2	Vasculogenesis/angiogenesis, vascular permeability, potential vasodilatation	*FLT1* *KDR*	TivozanibInhibitor Cabozantinib (Synthetic inhibitor)
28	Signalling events mediated by Hepatocyte Growth Factor Receptor (c-Met)	Cancer angiogenesis and cell invasiveness, hepatocyte regeneration	*HGF* *HGFR*	
29	EPO signalling pathway	Red blood cell production, implicated in hypertension	*EPO* *EPOR*	Peginesatide (Peptide agonist)
30	ATF-2 transcription factor network	Activating transcription factor	*ATF2*	
31	Signalling events mediated by focal adhesion kinase	Regulates hepatic stellate cell activation and liver fibrosis	*PTK2*	
32	Genes related to chemotaxis	Chemotaxis		
33	CDC42 signalling events	Cell morphology, cell migration, and cell cycle progression, implicated in liver regeneration	*CDC42*	
34	Glucocorticoid receptor regulatory network	Regulator of transcription		
35	Sphingosine-1-phosphate (S1P1) pathway	Immune response, vasculogenesis, vasodilatation, protective role in the liver	*S1PR1*	Siponimod (Synthetic Agonist)
36	Coregulation of androgen receptor activity	Regulator of transcription	*AR*	Mifepristone (Synthetic antagonist)
37	LPA receptor mediated events	Implications in inflammation, thrombosis and atherosclerosis	*LPAR1* *LPAR4* *LPAR6*	
38	Nongenotropic androgen signalling	Implications in hepatocellular carcinoma, cirrhosis, and hepatitis	*AR*	Mifepristone (Synthetic antagonist)
39	SHP2 signalling	Protects endothelial barrier, indirect role in vasodilatation through activation of eNOS	*PTPN11*	
40	IL5-mediated signalling events	Immune response, B-cell growth and differentiation, vascular cell growth and migration	*IL5* *IL5RA*	Mepolizumab Benralizumab (Monoclonal antibody)
41	HIF-2-alpha transcription factor network	Angiogenesis, regulates hepatocyte cell proliferation during hepatic outgrowth	*HIF1A*	
42	Syndecan-2-mediated signalling events	Cell proliferation, cell migration and cell–matrix interactions, vascular permeability	*SDC2*	
43	Validated targets of C-MYC transcriptional activation	Transcription factor	*MYC*	
44	Fas signalling pathway	Programmed cell death, role in liver cirrhosis transition to hepatocellular carcinoma	*FAS*	
45	Role of Calcineurin-dependent NFAT signalling in lymphocytes	Immune response	*PPP3CC*	
46	DNA-PK pathway in nonhomologous end joining	Potential role in alcohol-related liver disease	*PRKDC*	
47	Sumoylation by RanBP2 regulates transcriptional repression	Nuclear pore protein	*RANBP2*	
48	STAT3 Pathway	Transcription factor, regulator of liver fibrosis	*STAT3*	
49	Direct p53 effectors	Tumour suppressor gene, with potential roles in vascular remodelling and atherosclerosis	*P33*	

Principal gene(s) in the 49 pathways were compared against a curated list of genes encoding proteins to identify further potential targets in PH from the GuidetoPharmacology database (https://www.guidetopharmacology.org/) [44] that are currently unexploited. An example of a clinically approved drug is shown where these have been identified from the database, together with its drug class and mechanism of action.

### β-Adrenoceptor antagonists: α- and β-adrenoceptor receptor pathway

Analysis of differentially expressed genes did not identify any adrenergic signalling pathways as being significant. This is consistent with 40% of patients failing to respond to β-adrenoceptor antagonists, the most widely used agents for treating portal hypertension. Interpretation is complicated by six out of twelve of the patients being treated with these antagonists, that have altered expression of these genes. We therefore examined the expression of the genes encoding this pathway: six α- and three β-adrenoceptor sub-types that can be blocked by the clinically relevant drug carvedilol.

**Figure 5:**
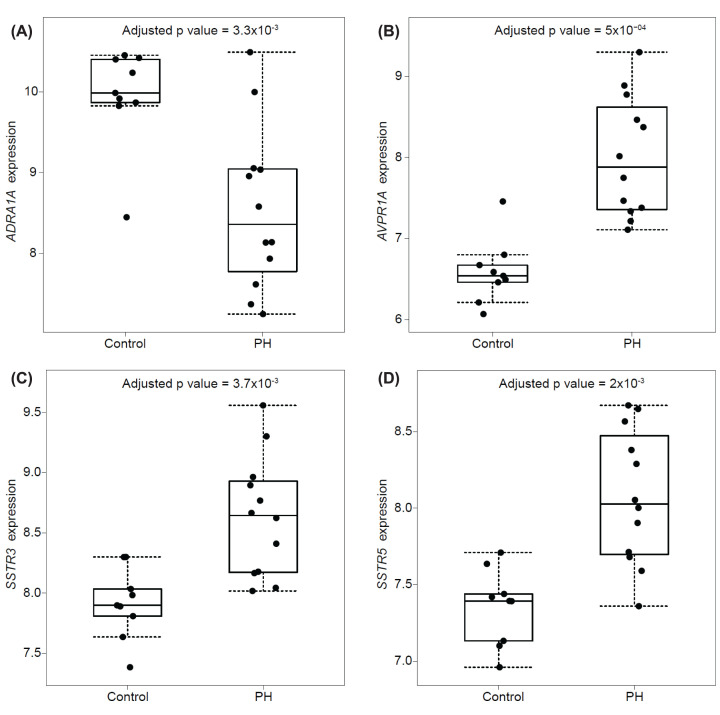
Expression of genes encoding targets of currently approved drugs that are differentially expressed in cirrhotic (PH) compared with control portal veins (**A**) Adrenoceptor α 1A *ADRA1A* was down-regulated, whilst (**B**) vasopressin receptor 1A gene *AVPR1A*, (**C**) somatostatin receptor 3 *SSTR3*, and (**D**) somatostatin receptor 5 *SSTR5* were all up-regulated in cirrhotic (PH) portal vein.

Vascular smooth muscle cells can express all six α-adrenoceptor sub-types [44], although in portal vein only *ADRA1A* (encoding α_1A_-adrenoceptor) and *ADRA2C* (α_2C_-adrenoceptor) were detected, the former down-regulated in PH (Figure 5A and Table 5). In agreement, phenylephrine, a full agonist targeting the α_1A_-adrenoceptor (also activates α_1B_ and α_1D_) caused a dose-dependent vasoconstrictor response (Figure 4C,D). Genes (*ADRB1, ADRB2, ADRB3*) encoding the three β-adrenoceptors (β_1_ β_2_ and β_3_) were not detected in portal vein in these samples. This is consistent with the mechanism of action of nadolol and timolol in β1 adrenoceptor blockade resulting in reduced cardiac output and reduced splanchnic blood flow. β2 receptor blockade results in splanchnic vasoconstriction, caused by the unopposed effect of α1 receptors, indirectly reducing PH, rather than a direct action on portal vein. Carvedilol, which is superseding propranolol, also has non-selective β-adrenergic receptor antagonist actions with additional vasodilating actions through α_1_ receptor blockade. This reduces porto-collateral resistance and, through effects on hepatic stellate cells, reduces intrahepatic resistance. The compound has additional antioxidant, antifibrotic, and anti-inflammatory properties, and may improve mitochondrial function and enhance insulin sensitivity.

**Table 5:** Approved drugs in the treatment of PH

Drug	Target	Expressed	Differentially expressed	Route of administration	Receptor	Reference
Terlipressin	*AVPR1A* *AVPR1B* *AVPR2* *Ligand* *AVP*	Yes No Yes No	Yes ↑ No No No	Intravenous	V_1A_ V_1B_ V_2_	[73]
Octreotide	*SSTR2, SSTR3, SSTR5* *Ligand* *SST*	No Yes Yes No	No Yes↑ Yes↑ No	Intravenous	SST_2_ SST_3_ SST_5_	[74,75]
Propranolol	*ADRA1A* *ADRA2A* *ADRA2B* *ADRA2C* *ADRB1* *ADRB2* *ADRB3*	Yes No No Yes No No No	Yes↓ No No No No No No	Oral	α_1A_-adrenoceptor α_2A_-adrenoceptor α_2B-_adrenoceptor α_2C_-adrenoceptor β_1_-adrenoceptor β_2_-adrenoceptor β_3_-adrenoceptor	[76]
Nadolol	*ADRB1* *ADRB2* *ADRB3*	No No No	No No No	Oral	β_1_-adrenoceptor β_2_-adrenoceptor β_3_-adrenoceptor	
Timolol	*ADRB1* *ADRB2* *ADRB3*	No No No	No No No	Oral	β_1_-adrenoceptor β_2_-adrenoceptor β_3_-adrenoceptor	
Carvedilol	*ADRA1A* *ADRA1B* *ADRA1D* *ADRA2A* *ADRA2B* *ADRA2C* *ADRB1* *ADRB2* *ADRB3*	Yes No No No No Yes No No No	Yes↓ No No No No No No No No	Oral	α_1A_-adrenoceptor α_1B_-adrenoceptor α_1D_-adrenoceptor α_2A_-adrenoceptor α_2b_-adrenoceptor α_2c_-adrenoceptor β_1_-adrenoceptor β_2_-adrenoceptor β_3_-adrenoceptor	

The genes encoding protein targets of currently approve drugs to treat PH from the GuidetoPharmacology database (https://www.guidetopharmacology.org/) [44]. These are compared with the list of genes that were expressed in portal veins and whether these are significantly differentially up- or down-regulated between cirrhotic and control portal veins.

### Identification of existing pharmacological targets in PH to validate the microarray strategy

The microarray identified up-regulation of G protein-coupled receptor genes in two pathways that are currently exploited in PH in the setting of acute variceal haemorrhage and hepatorenal syndrome.

### Terlipressin: vasopressin pathway

The vasopressin receptor genes *AVPR1A* (V_1A_ receptor) and *AVPR2* (V_2_ receptor), but not the ligand, were detected, with the former up-regulated in PH (Figure 5B and Table 5). *AVPR1A* was one of the genes displaying the highest fold change. Expression is consistent and provides an elegant mechanistic explanation for the use of terlipressin, which acts as a vasopressin agonist in patients with end-stage liver disease to increase systemic vascular resistance through direct actions on V_1A_ receptors in smooth muscle to cause vasoconstriction. This occurs particularly in the splanchnic area, resulting in a decrease of portal pressure. V_2_ receptors are expressed on endothelial cells and beneficially indirectly release vasodilators as a normal physiological counter-regulatory process [8].

### Octreotide: somatostatin pathway

Octreotide is a nonspecific somatostatin analogue with a long half-life, acting on somatostatin SST_2_, SST_3_, and SST_5_ receptor sub-types. It has a similar pharmacological action to terlipressin, acting on receptors in the splanchnic circulation to cause vasoconstriction and reduce portal hypertension. Genes encoding the SST_3_ (*SST3*) and SST_5_ (*SST5*) receptors were both up-regulated (Figure 5C,D and Table 5) in PH portal vein but the gene encoding the peptide ligand was not [8]. In a similar manner to terlipressin, this also provides a mechanistic explanation for the efficacy of octreotide in PH and provides additional validation of the potential to identify therapeutic targets.

## Discussion

PH is defined as an increased pressure within the portal venous system with cirrhosis of the liver a common cause. Current drug therapy to reduce portal pressure is mainly limited to β-adrenergic receptor blockade but approximately 40% of patients do not respond indicating a need for new targets and therapeutics.

A microarray was used to measure ∼20,800 genes in surgical samples of portal veins (Figure 1) from patients undergoing liver transplantation for PH caused by cirrhosis versus controls. As proof-of-concept, three genes encoding G protein-coupled receptors (*SST3*, *SST5*, and *AVPR1A*) that are current therapeutic targets in PH in the setting of acute variceal haemorrhage and hepatorenal syndrome were up-regulated (Figure 5).

In this study, pathway analysis showed that the ET pathway was significantly enriched for genes differentially expressed between portal vein from patients undergoing liver transplantation for PH caused by cirrhosis and controls. The ET-1 gene was significantly up-regulated (Figure 3A), consistent with the peptide contributing to the imbalance of constrictor tone in PH. While the gene encoding the ET_A_ receptor was significantly down-regulated (Figure 3B), functional assays in portal vein from a patient with PH still responded to ET-1 induced constriction (Figure 4), which was fully blocked by an approved ET_A_ receptor antagonist, ambrisentan, at a clinically relevant concentration. There was no significant change in the gene encoding ET_B_ (Figure 3C) suggesting the vasodilator response would be beneficially maintained. Pathway analysis identifies biological pathways that are enriched in differentially expressed genes more than would be expected by chance. It provides additional evidence that blocking ET constriction of the portal vein using ET_A_ receptor antagonists could be a potentially effective therapy in patients with PH as a consequence of cirrhosis.

ET receptor antagonists that block both ET_A_ and ET_B_ receptors have been used to treat pulmonary arterial hypertension (PAH) since bosentan was first approved for clinical use in 2002 [26]. However, this mixed antagonist is contraindicated in patients with hepatic function impairment as a result of a risk of hepatotoxicity and raised transaminases [53]. A second generation approved mixed antagonist, macitentan, also has similar limitations in liver disease, although Kim and colleagues [54] have reported, in patients with portopulmonary hypertension and liver disease, that this drug was safe with few side effects.

The development of hepatotoxicity by mixed ET_A_/ET_B_ antagonists has been linked to ET_B_ receptor antagonism, which causes modulation of hepatobiliary transporters such as bile salt export, causing an accumulation of cytotoxic bile acids [55]. A possible mechanism is suggested by selective knocking out of endothelial ET_B_ receptors in mice, with sinusoids markedly reduced in both number and absolute diameter, while large intrahepatic veins were congested with red blood cells. ET_B_ blockade may cause portal sinusoid constriction and further cholestasis [56].

A key proof-of-principle study showed that, in patients with PH, there was a significant decrease of the portal pressure estimated by HVPG with the administration the highly selective ET_A_ peptide antagonist BQ123 [57] into the portal vein. Systemic administration of ambrisentan also caused a reduction in HVPG, independent of changes in systemic haemodynamics. An increase in hepatic arterial flow was also independent of the changes in systemic circulation, consistent with a local action of ET_A_ receptor blockade [58].

Two earlier clinical studies have also measured the acute effects of HVPG in patients with PH. The ET_A_ /ET_B_ antagonist tezosentan (at a dose of 3 mg/h for 2–3 h) did not alter HVPG but, surprisingly, there was also no expected change in arterial pressure in either patients with PH or volunteer controls, suggesting low or no efficacy of the compound at this concentration [59]. Similarly, Tripathi and colleagues infused BQ123 into a small number of patients (*n*=8), at 1000 and 3000 nmol/min that also had no short-term action on HVPG or systemic vascular resistance index [60].

Long-term treatment with ambrisentan demonstrated a low risk of aminotransferase abnormalities in pulmonary arterial hypertension patients [61]. However, a planned phase II study (NCT03827200) using ambrisentan on portal pressure in patients with advanced liver cirrhosis and with PH was terminated early owing to lack of recruitment resulting from the COVID-19 pandemic [62]. Two recent drug approvals have potential for repurposing in PH. Clazosentan, a highly selective ET_A_ receptor antagonist, has been approved for the prevention of cerebral vasospasm following aneurysmal subarachnoid hemorrhage [63], but a limitation is that it is an intravenous drug. Sparsentan is a dual-acting selective ET_A_ and angiotensin II receptor type 1 receptor (AT_1_) antagonist approved in 2023 for the treatment of patients with IgA nephropathy [64]. Remarkably, sparsentan combines high nanomolar affinity for ET_A_ and sub-nanomolar affinity for AT_1_ in the same molecule. Combination in the same molecule maximizes efficacy with more predictable receptor occupancy, avoiding mismatched pharmacokinetics and variability in metabolism, improving patient compliance [26]. Although not extensively studied in clinical trials, the main beneficial action of blocking the renin–angiotensin pathway in patients with cirrhosis and PH is to reduce fibrosis [65].

A major limitation to the use of ET antagonists has been unwanted side effects, particularly fluid retention. For example, peripheral edema was reported in 17% of patients with PAH treated with bosentan [66]. A serendipitous discovery was made in a trial where atrasentan alone resulted in increased body weight by more than 1 kg, indicating fluid retention. In contrast, a small number of patients that were also treated with a sodium-glucose co-transporter-2 inhibitor (SGLT2i) showed a decrease in body weight [67]. Further evidence supporting this hypothesis included a preclinical study demonstrating that the SGLT2i, dapagliflozin, prevented hemodilution and increases in body weight in salt fed rats treated with the highly selective ET_A_ antagonist, zibotentan [68]. This provided the rationale for a phase IIb study of zibotentan with dapagliflozin in treatment of chronic kidney disease and, as proof-of-principle, showed that zibotentan induced fluid retention was partly prevented by co-administration of dapagliflozin [69]. SGLT2i monotherapy in patients with liver cirrhosis has shown beneficial actions [70] potentially as a result of improved endothelial function, decreased inflammation and improved fluid balance. A phase IIa/b multicentre study (https://clinicaltrials.gov/ct2/show/NCT05516498) has commenced comparing zibotentan and dapagliflozin in combination with dapagliflozin monotherapy versus placebo in patients with cirrhosis and features of PH to assess the effects of endothelin receptor/SGLT2 combinatorial drug strategy [71].

The current pharmacological treatment mainly aims at reducing splanchnic blood flow and/or hepatic resistance [73,74,75,76]. Many patients with PH do not respond to targeting splanchnic blood flow and/or hepatic resistance, and new targets for new treatments need to be identified. However, while the portal vein itself is not the sole contributor to the development of PH, evidence from animal models suggests that ET-1 may be important in causing portal vasoconstriction [35], and it is not necessarily all attributable to portal vein inflow (splanchnic vasodilatation) or intrahepatic resistance.

In conclusion, the results of the present study provide further support for evaluating the efficacy of ET_A_ receptor antagonists as a potential therapy in addition to β-blockers in patients with PH and cirrhosis.

## Data Availability

Authors agree to make any materials, data, and associated protocols available upon request. Microarray data will be made available on Figshare on acceptance of manuscript.

## Open Access

Open access for this article was enabled by the participation of University of Cambridge in an all-inclusive *Read & Publish* agreement with Portland Press and the Biochemical Society under a transformative agreement with JISC.

## Abbreviations

ARLD: alcohol-related liver disease
FC: fold change
HVPG: hepatic venous pressure gradient
MASLD: metabolic dysfunction-associated steatotic liver disease
MELD: Model for End-Stage Liver Disease
PH: portal hypertension
UKELD: United Kingdom Model for End-Stage Liver Disease
